# LVPT: Lazy Velocity Pseudotime Inference Method

**DOI:** 10.3390/biom13081242

**Published:** 2023-08-12

**Authors:** Shuainan Mao, Jiajia Liu, Weiling Zhao, Xiaobo Zhou

**Affiliations:** 1 The Department of Biotherapy and West China Biomedical Big Data Center, West China Hospital, Sichuan University, Chengdu 610041, China; 2 Med-X Center for Informatics, Sichuan University, Chengdu 610041, China; 3 Center for Computational Systems Medicine, McWilliams School of Biomedical Informatics, The University of Texas Health Science Center at Houston, Houston, TX 77030, USA; 4 McGovern Medical School, The University of Texas Health Science Center at Houston, Houston, TX 77030, USA; 5 School of Dentistry, The University of Texas Health Science Center at Houston, Houston, TX 77054, USA

**Keywords:** single cell, trajectory inference, pseudotime inference, random walk

## Abstract

The emergence of RNA velocity has enriched our understanding of the dynamic transcriptional landscape within individual cells. In light of this breakthrough, we embarked on integrating RNA velocity with cellular pseudotime inference, aiming to improve the prediction of cell orders along biological trajectories beyond existing methods. Here, we developed LVPT, a novel method for pseudotime and trajectory inference. LVPT introduces a lazy probability to indicate the probability that the cell stays in the original state and calculates the transition matrix based on RNA velocity to provide the probability and direction of cell differentiation. LVPT shows better and comparable performance of pseudotime inference compared with other existing methods on both simulated datasets with different structures and real datasets. The validation results were consistent with prior knowledge, indicating that LVPT is an accurate and efficient method for pseudotime inference.

## 1. Introduction

Cells, as the fundamental building blocks of life, prompt fundamental inquiries into their growth and development. With the development of high-throughput sequencing technology, single-cell sequencing technology can provide both mRNA expression values and spliced and unspliced gene expression values, which provides more possibilities for studying cell development and evolution. However, due to the limitations of experimental technologies, continuous changes in gene expression values cannot be observed during cell differentiation. Therefore, inferring cell differentiation pseudotime and trajectory from discrete data stands as a burgeoning research focus in single-cell analysis.

Numerous machine learning methods have been used to construct trajectories to tackle the aforementioned issue [1,2,3,4,5,6]. These methods can be classified into two distinct categories: dimensionality-reduction-based methods and K-Nearest Neighbor (KNN) graph-based methods. The methods based on dimensionality reduction include Monocle [7,8,9,10], Slingshot [11], Waterfall [12], and STREAM [13]. Monocle introduced a pioneering trajectory inference model that encompasses multiple steps, including preprocessing, dimensionality reduction, clustering, minimum spanning tree construction, principal curve fitting, and projection to form single-cell differentiation trajectory trees. Monocle’s sensitivity to parameter settings can make it challenging to achieve consistent results across different datasets. Slingshot further extended the scope by allowing greater flexibility in the selection of dimensionality reduction and clustering algorithms. It used the Mahalanobis-like distance when constructing the minimum spanning tree. The Mahalanobis-like distance approach in minimum spanning tree construction, while innovative, could be sensitive to outliers, potentially affecting the overall trajectory structure. STREAM used an improved Locally Linear Embedding (LLE) algorithm for dimensionality reduction and then used a greedy optimization process to infer the principal curve. The improved LLE algorithm in STREAM may introduce computational complexity, particularly when applied to extensive datasets. The KNN graph-based methods include DPT [14], scTDA [15], PAGA [16], etc. DPT used a random walk model to calculate the average access time as pseudotime and detect the branch structure. The method scTDA used Topology Data Analysis to build the cell fate graph. PAGA used an abstract graph to model the structure of cells. The KNN graph-based methods are sensitive to the choice of the k parameter. Balancing the trade-off between capturing local versus global relationships becomes critical, and finding the optimal k value can be challenging.

Pseudotime inference changes with the development of technology. At the early stage of single-cell development, most models can only make linear inference. There are many methods based on various statistical models, such as DeLorean [17], which used the Gaussian Process Latent Variable Model, CSHMM [18], which used the Hidden Markov Model, and Wanderlust [19], which used shortest distance on the graph to calculate the pseudotime. With the continuous development of single-cell trajectory inference, pseudotime inference is gradually divided into two categories. One type is projection-based methods, which use cells to project onto the latent backbone and calculate the distances between cells and the root cell as pseudotime, typically Monocle and Slingshot. The other type is random-walk-based methods, which sort cells by calculating the hitting time of random walks on the graph, typically methods such as DPT, PAGA, VIA [20], and VPT [21]. PAGA used DPT to calculate pseudotime after constructing abstract graphs. The VIA method performed random walk at the clustering level and introduced the concepts of inertia and transport to increase robustness. The concept of RNA velocity [22] was proposed to integrate information about the transcription process. VPT applied RNA velocity to the pseudotime inference. There are some other methods for pseudotime inference, such as Waddington-OT [23] and LineageOT [24], which use the optimal transport method for time series data, TinGa [25], which uses a neural network, and Topographer [26]. Although numerous methods have been diligently proposed to address the challenges of pseudotime inference, the complex and multifaceted nature of single-cell data presents an ongoing frontier for exploration, and new models are needed to achieve better results.

The introduction of RNA velocity has taken the research on pseudotime inference to a new stage. Although some algorithms such as VPT have been proposed, there is still room for further exploration. Here, inspired by VIA, we proposed LVPT (Lazy Velocity Pseudotime), a novel pseudotime inference model. LVPT uses the Gaussian kernel function as the distance between cells and integrates RNA velocity into pseudotime inference to provide information about the direction of cell differentiation. A lazy probability is introduced in LVPT to indicate the probability that the cell will remain in that state during random walking due to cell division. In addition, we also construct a complete single-cell trajectory inference model to analyze the overall cell evolution. Our experiments show that LVPT outperforms other methods in terms of accuracy on the simulated datasets and the predicted cell evolution process is accurately restored in the real datasets.

## 2. Materials and Methods

### 2.1. Overview

Currently, there are many KNN graph-based methods, such as VPT and VIA. Although these methods have achieved good results, they all have their own limitations. Our method, LVPT, solves the following problems on the basis of existing methods. The first is to construct a biological model of random walk, which provides a solid theoretical foundation for introducing inert probability methods. Compared with VPT, LVPT can better reduce the process of cell changes and has higher robustness. Compared with VIA, LVPT explains the inertia probability and migration probability in random walk from the perspective of cell division and differentiation. In our hypothesis, we did not consider gene mutations, so we abandoned the concept of transmission in VIA to make the model more rigorous. Secondly, unlike VIA performing random walk at the clustering level, LVPT performs random walk at the cellular level, which can utilize more information. Thirdly, after introducing the concept of rate, we can use Markov processes to determine the root node of the trajectory instead of it being manually set by the user like in VIA.

### 2.2. Data Collection and Preprocessing

In LVPT, we used the mRNA expression matrix and spliced and unspliced expression matrix of scRNA-seq data as the input. Spliced and unspliced data can be calculated using the tools provided in the RNA Velocity [22]. The root cell can be provided by users as priori data or calculated from expression data. We experimentally validated the method with datasets of mouse pancreas [27] and mouse hippocampus [22]. Sequencing data need to be properly preprocessed. We set a minimum threshold for expression counts, then centered and normalized the data. By calculating the dispersion of each effective gene, we took highly variable genes as filtered genes. Finally, we converted the expression data from count data to log-transformed data to facilitate further analysis. We set the minimum expression value to 20 and selected 2000 genes for the experiments in this paper.

### 2.3. Data Simulation Using Dyngen

We used dyngen, a multi-modal simulation engine for studying dynamic cellular processes at a single-cell resolution [28], to generate simulated data for testing the effectiveness of the algorithm. The simulated data contain 1000 cells and 100 expressed genes which were generated with the GillespieSSA2 method with the parameter τ = 1/12. Different simulated datasets were obtained by setting linear, bifurcating, and trifurcating trajectory backbones for the evaluation of LVPT. The simulated datasets contain spliced and unspliced expression read counts to calculate cell velocity. The simulated datasets provide the ground truth of pseudotime which can be used as a gold standard for comparison with the predicted pseudotimes.

### 2.4. Lazy Velocity Pseudotime Inference Model

We developed the LVPT model to infer the cell states, including pseudotime and trajectory. LVPT consists of four modules, including clustering, velocity estimation, pseudotime inference, and trajectory inference. For clustering, we used the Leiden [29] algorithm to obtain cell clusters as trajectory nodes. By modeling the kinetic of the gene transcription process, the relationship between the expression of unspliced mRNA and spliced mRNA can be used to infer the amount of change in the gene expression value in the future, known as cell velocity. We used the *scvelo.tl.velocity* [21] function to estimate cell velocity. The basic idea of velocity estimation is to model the transcription process of cells and calculate the trend of gene expression changes in cells over a short period of time in the future using spliced and unspliced expression values. The root cell can then be inferred using the *scvelo.tl.terminal_states* [21] function. The function models dynamic cellular processes as a Markov chain, where the transition matrix is computed based on the velocity vector of each individual cell. Based on this Markov chain, cells are filtered into transient/recurrent cells using the left eigenvectors of the transition matrix and clustered into distinct groups of terminal states using the right eigenvectors of the transition matrix of the Markov chain [21]. The root cells are obtained as stationary states of the transpose of the velocity-inferred transition matrix, which is given by left eigenvectors corresponding to an eigenvalue of 1, i.e.,

(1) $$\mu^{root}=\mu^{root}\pi^{T}$$

where $\mu^{root}$ is the left eigenvectors and *π* is the velocity-inferred transition matrix in LVPT. The main contribution of this study is to provide a new pseudotime inference method. We integrated the estimated velocity with the information from spliced and unspliced data to infer the pseudotime in this model, making pseudotime inference more accurate. Finally, we also analyzed the transition relationship between cell states at cluster resolution to construct cell differentiation trajectories. Partition-based graph abstraction (PAGA) is a reliable method for constructing the connections between cell states [16]. PAGA uses the statistical model to calculate the connection possibility between clusters and constructs one or more undirected graphs. We first used PAGA to construct an abstract trajectory graph, then applied the average pseudotime of cell states to judge the transition direction between states and finally constructed a directional trajectory structure.

### 2.5. Pseudotime Inference

Cell growth implies the sequential expression of different genes, including several different intermediate steady stages [30,31]. We model this process using cell division and differentiation. Cell division means the cell is cloning itself, the expression level is in a steady stage, and cell differentiation means the cell is evolving from one stage to another, the expression level is going to another steady stage. The process of cell division and differentiation is random. We can simulate it through the random walk method. Briefly, if a cell goes through cell division to the next step, the expression should not be changed; otherwise, it is changed. The core of the random walk model is the transition probability matrix. We constructed a reliable random walk model by introducing lazy probability and cell velocity.

At the cell level, cell steady-stage expression is not a constant value; it can be affected by noises. We use the *G* function to model gene expression distribution. The transition probability from cell *i* to cell *j* in a steady state is defined as

(2) $$P_{1}(x_{j}|x_{i})=G(x_{j}|x_{i},\sigma_{i})$$

(3) $$\sigma_{i}=\left|x_{i}-x_{k}\right|$$

where *G* means Gaussian function, and $P_{1}$ represents the probability that the cell is in its current state, not through differentiation but through self-proliferation or external influences that alter its expression. The center of the function is at the expression value $x_{i}$ of the current cell *i*. The variance $\sigma_{i}$ is the Euclidean distance in expression space between the cell *i* and its *k*th neighbor, where *k* is a parameter when generating the neighbor network. In this way, each cell possesses a distinct Gaussian kernel function that preserves information within the cell’s local neighborhood.

We define the transition probability during differentiation as

(4) $$P_{2}\left(x_{j}|x_{i},\sigma_{i},\delta_{ij}\right)=\left\{\begin{array}{c} \delta_{ij}G\left(x_{j}|x_{i},\sigma_{i}\right),\;\;\delta_{ij}\geq 0,\\ 0,\;\;\delta_{ij}<0, \end{array}\right.$$

(5) $$\delta_{ij}=\cos(v_{i},x_{j}-x_{i})$$

where $\delta_{ij}$ is the cosine value between the cell velocity $v_{i}$ and the vector composed of cells $x_{i}$ and $x_{j}$, which represents the cell velocity component in the direction of the vector composed of the target cell *j* and the current cell *i*. The probability $P_{2}$ indicates that if the $\delta_{ij}$ is positive, the closer the cell *j* is to the cell velocity direction $v_{i}$, the greater the probability of migrating to the target cell *j*; if the $\delta_{ij}$ is negative, the target cell is located in the opposite direction of the differentiation process and should not migrate to the target cell j, and the transition probability should be 0.

The complete transition probability of a cell is defined as

(6) $$P=\alpha P_{1}+\left(1-\alpha \right)P_{2}$$

where *α* is the lazy probability, which indicates that the cell will walk to the next stage through division. After regularization, we can obtain the cell-to-cell transition probability matrix:

(7) $$T^{asym}=\frac{P\left(x,y\right)}{\sum_{y\in y}P\left(x,y\right)}$$

where *x* and *y* are the cells. The original diffusion map publication [32] pointed out that there is a symmetric matrix *T* that has the same eigenvalues as asymmetric matrix *T*:

(8) $$T=Z{\left(x\right)}^{-\frac{1}{2}}P\left(x,y\right)Z{\left(y\right)}^{\frac{1}{2}}$$

(9) $$Z\left(x\right)=\sum_{y\in \Omega }P\left(x,y\right)$$

After determining the transition probability matrix, we can calculate the pseudotime $t=\left\{t_{1},\ldots ,t_{n}\right\}$ result with the random walk [14]. LVPT uses the same random walk computational method with DPT. $f\left(t\right)$ is defined as the reaching probability from $f\left(0\right)$ in time *t*:

(10) $$f\left(t\right)=f\left(t-1\right)T=f\left(0\right)T^{t}$$

By summarizing the probabilities, we can obtain the (time independent) “path integral” for reaching each cell from $f\left(0\right)$:

(11) $$\sum_{t=1}^{\infty }f\left(t\right)=f\left(0\right)\sum_{t=1}^{\infty }T^{t}$$

$f\left(0\right)$ is initialized by root cells. By reducing the stationary component to make the sum above converge, we obtain a new matrix *M*:

(12) $$\begin{array}{ll} M\left(x,z\right) & =\sum_{t=1}^{\infty }{\left(T\left(x,z\right)-\psi_{0}\left(x\right)\psi_{0}^{T}\left(z\right)\right)}^{t}\\ & =\sum_{i=1}^{n-1}\left(\frac{\lambda_{i}}{1-\lambda_{i}}\right)\psi_{i}\left(x\right)\psi_{i}^{T}\left(z\right) \end{array}$$

If the random walk starts at cell *x*, $f\left(0\right)M$ will be a row of *M* which we present by $M\left(x,.\right)$. Then we can define the lvpt measure as

(13) $$\begin{array}{ll} lvpt^{2}\left(x,y\right) & =|M\left(x,.\right)-M(y,.){\left.\right|}^{2}\\ & =\sum_{z}{\left(M\left(x,.\right)-M\left(y,z\right)\right)}^{2}\\ & =\sum_{i=1}^{n-1}{\left(\frac{\lambda_{i}}{1-\lambda_{i}}\right)}^{2}{\left(\psi_{i}\left(x\right)-\psi_{i}\left(y\right)\right)}^{2} \end{array}$$

The pseudotime of cell *x* from root *r* is

(14) $$lvpt\left(x,r\right)$$

### 2.6. Evaluation Metrics

Saelens et al. established a complete evaluation framework [33] for single-cell trajectory inference. Based on this framework, we defined the correlation between the estimated pseudotime and the true value using the Spearman correlation coefficient. The testing dataset generated with the single-cell simulation method provides us with a real-time view of each cell. If the real time is defined as $t^{r}$ and the inferred pseudotime is defined as *t*, the Spearman coefficient can be defined as follows:

(15) $$\rho \left(t^{r},t\right)=\frac{\sum_{i}\left(t_{i}-\bar{t}\right)\left(t_{i}^{r}-\bar{t^{r}}\right)}{\sqrt{\sum_{i}{\left(t_{i}-\bar{t}\right)}^{2}\sum_{i}{\left(t_{i}^{r}-\bar{t^{r}}\right)}^{2}}}$$

The Spearman correlation coefficient indicates the correlation between *t* and $t^{r}$. When using the real time and the inferred pseudotime as the parameters of the Spearman correlation coefficient, the Spearman coefficient can be used as the accuracy evaluation index of the result. The closer the accuracy is to 1, the better the inferred result of the model is.

The Hamming–Ipsen–Mikhailov (HIM) metric is used to compare the trajectories. Conceptually, the HIM metric is a linear combination of the normalized Hamming distance and the normalized Ipsen–Mikhailov distance. The Hamming distance calculates the distance between two graphs by matching individual edges in the adjacency matrix but disregards overall structural similarity. The Ipsen–Mikhailov distance calculates the overall distance of two graphs based on matches between its degree and adjacency matrix, while disregarding local structural similarities. It requires a parameter, which we fixed at 0.1 to make the score comparable across different graph sizes.

## 3. Results

### 3.1. Overview

**Figure 1:**
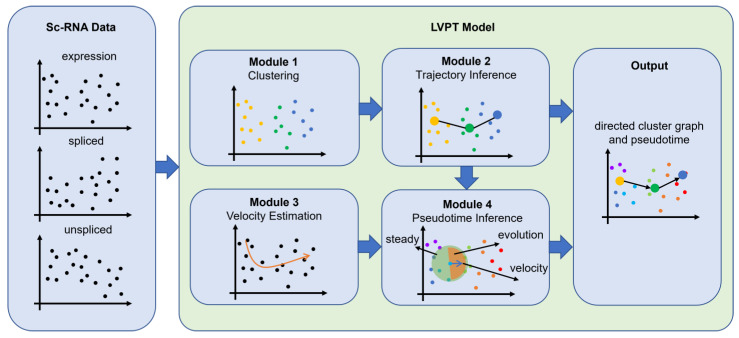
Overall workflow of the LVPT model.

As shown in Figure 1, the input into the LVPT model includes the gene expression matrix for a given starting node, the spliced gene expression matrix, and the unspliced gene expression matrix from scRNA-seq data. The LVPT model consists of four modules, including clustering, velocity estimation, pseudotime inference, and trajectory inference. We developed a new method for the pseudotime inference module. In this module, we used cell velocity and the gene expression matrix to calculate pseudotime during cell growth and development, the Leiden algorithm to cluster the cells, scVelo for velocity estimation, and the PAGA method to obtain the connection relationship between clusters and combined this with pseudotime to determine the direction of trajectory connection.

### 3.2. Evaluation of the LVPT Model on Simulated Datasets

To verify the effectiveness of the LVPT model, we used simulated datasets to compare the accuracy of LVPT with other methods. First, we used a dyngen simulation to generate simulated data with different trajectory structures, including a linear structure, a bifurcating structure, and a trifurcating structure. Then, we used the dynmethods [33] library to call different algorithms, including LVPT, VPT, VeTra [34], CellPath [35], PAGA, Monocle2, Slingshot, DPT, and TSCAN. Five simulation datasets were generated for each structure. Using these datasets, we tested each method under the same experimental conditions. The average Spearman correlation between pseudotime and the ground truth over the five simulated datasets was used as the accuracy to evaluate the performance. The predicted accuracy and HIM distance are shown in Table 1.

**Figure 2:**
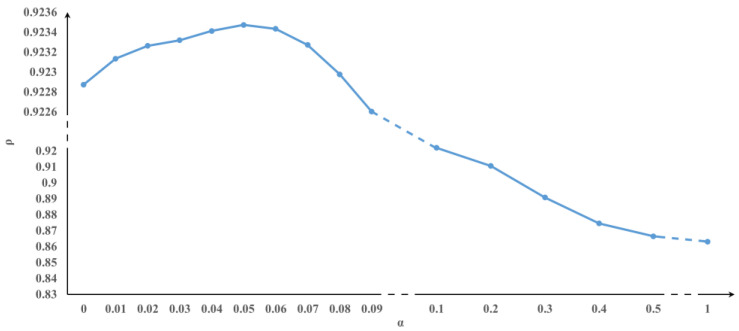
Variation in the accuracy of trifurcating simulated data under different α.

As can be seen from Table 1, the accuracy and HIM distance of LVPT are slightly higher than those of the other methods, especially in the case of complex branch structures. The results of LVPT outperform DPT. This is because we introduced the cell velocity into the transition matrix, which integrates the information about the transcriptional state of the cell and helps to judge the differentiation state of cells better. The accuracies of LVPT and VPT are similar. But our method employed a hyperparameter α to control the proportion of steady probability. Figure 2 shows that the tuning hyperparameters yield better accuracy. It also indicates that adding steady probability can improve the performance of pseudotime inference. However, when α is too high, the differentiation part in the transition probability formula will not have an effect, and the accuracy will be significantly reduced (See Figure 2). Thus, we recommend a reasonable range of hyperparameters to be 1–10% [20].

### 3.3. Performance Evaluation of LVPT on Real Datasets

The pancreatic tissue dataset was derived from mouse pancreatic endocrine cells. After processing the data, we obtained 3696 single-cell data points and 2000 genes as feature attributes.

**Figure 3:**
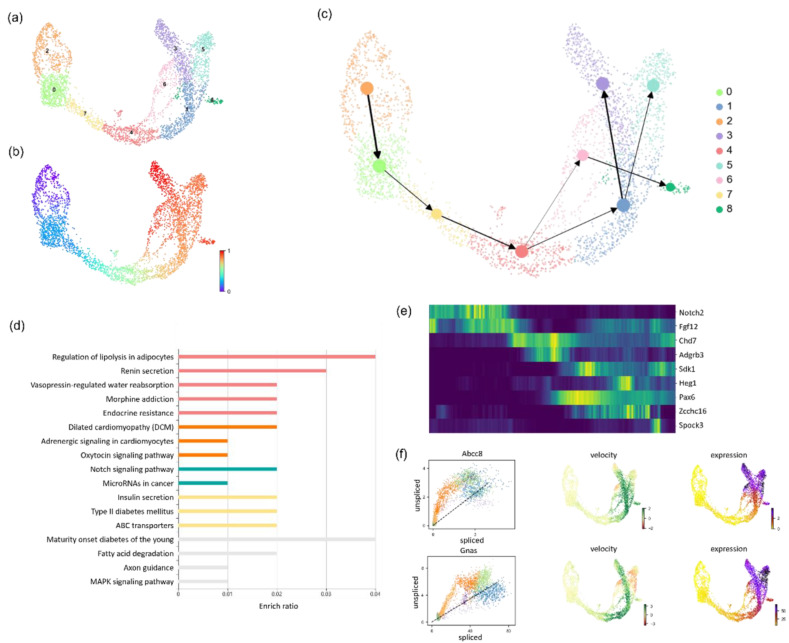
Application of LVPT to the pancreas dataset. (**a**) Scatter plot of the Leiden clustering result. (**b**) Scatter plot of pseudotimes inferred by LVPT. (**c**) Trajectory plot with clustering labels. The width of the edge indicates the weight between the clusters. (**d**) Histogram of enrichment analysis results. Different colors represent different categories. (**e**) Heatmap of differential genes. Genes are ordered by trajectory, and expression is ordered by pseudotime. (**f**) Spliced and unspliced expression plots, velocity plots, and mRNA expression plots of Abcc8 and Gnas.

After PCA processing, the 30 top-ranked principal components were selected as the features after dimension reduction, and the nearest neighbor network was constructed by setting the number of neighbors as 30 according to the data scale. We used the Leiden clustering algorithm to set the resolution parameter to 0.54, and the pseudotime inference hyperparameter α was equal to 0.05. The results are shown in Figure 3. After clustering, the cells were divided into nine clusters. It can be seen from the pseudotime diagram and trajectory diagram that the cells started to develop from cluster 2 and differentiated into clusters 3, 5, and 8.

This result indicates that pancreatic endocrine cells start to differentiate from pancreatic ductal epithelial cells, go through the process of endocrine progenitors, gradually differentiate into proendocrine cells, and finally differentiate into glucagon-producing alpha cells to produce insulin-producing beta cells, somatostatin-producing delta cells, and ghrelin-producing epsilon cells. We performed differential analysis on the clustering results to identify the genes with the highest differential expression in each category and arranged them in the order of trajectories.

We applied LVPT to the dataset of mouse hippocampus with a larger data volume and more complex topological structure. The mouse hippocampus dataset has 18,213 cell samples, and 2000 genes were selected as sample features after preprocessing. According to the sample size, we set the number of neighbors to 100 to construct the neighbor networks. We set the resolution parameter of the Leiden algorithm to 0.85 and obtained 12 clusters. α was set to 0.05 to calculate the pseudotime distribution and cell trajectory.

**Figure 4:**
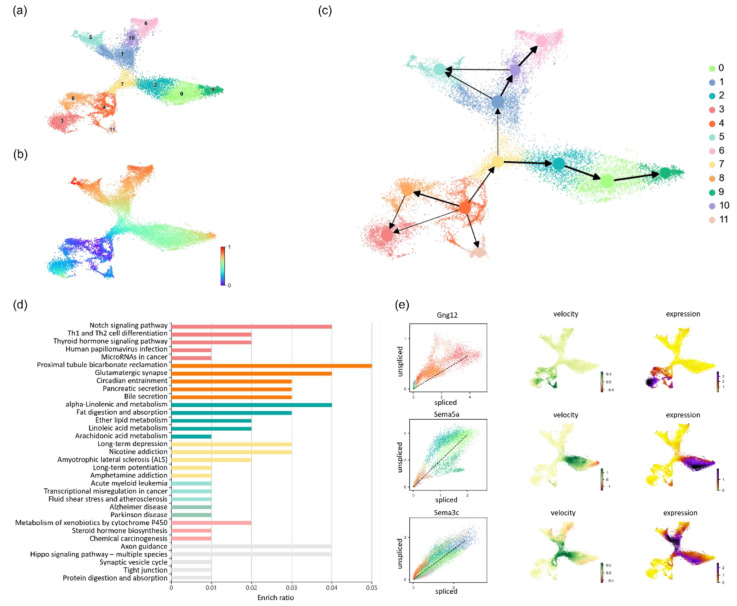
Application of LVPT to the hippocampus dataset. (**a**) Scatter plot of the clustering result. (**b**) Scatter plot of the pseudotime inferred by LVPT. (**c**) Trajectory result plot. The evolution of hippocampal cells starts from cluster 4 and moves toward 5 evolution directions. (**d**) Histogram of enrichment analysis results. Different colors represent different categories. (**e**) Spliced and unspliced gene expression plot, velocity plot, and mRNA expression plot of Gng12, Sema5a, and Sema3c.

As shown in Figure 4c, cells start to differentiate from cluster 4, the intermediate progenitor cells, and move toward three evolutionary directions. First, through narrow channels, intermediate progenitor cells differentiate into oligodendrocyte precursors (cluster 11). Second, through intermediate radial glial transformation, intermediate progenitor cells differentiate into astrocytes (cluster 3). Third, intermediate progenitor cells are evolved into Nbl2 cells (cluster 7). In the third evolutionary direction, the differentiated Nbl2 cells face another fate choice, that is, to differentiate into dentate gyrus granule neurons (cluster 9) or CA cells (cluster 1). CA cells then differentiate into CA1-CA4 and subtotal cells (clusters 5, 6, 10), while dentate gyrus granule neurons develop into mature cells after passing through two intermediate states. Our results are consistent with major functional and anatomical subdivisions of the hippocampus, validating the effectiveness of LVPT [36].

Through gene enrichment analysis, we found the axon guidance signaling pathway (KEGG number mmu04360) and analyzed the data distribution of its related genes Gng12, Sema5a, and Sema3c. As shown in Figure 4e, these genes have different regulatory roles. The Gng12 gene is mainly distributed in the glial cell area, the Sema5a gene is mainly related to the formation of dentate gyrus granule neurons, and Sema3c mainly affects the initial CA cells and dentate gyrus granule neurons.

## 4. Discussion

The development of single-cell sequencing technology has played an important role in understanding the life course of cells. With the continuous development of technology, the potential of single-cell sequencing data is constantly being explored. Early studies directly used dimension reduction methods or topological distances for trajectory inference on mRNA expression data. With the deepening of research, more complex and improved models have been continuously proposed, and data during transcription process have also been mined and integrated into RNA velocity, providing us with more useful information. LVPT presents a simple and efficient method for trajectory and pseudotime inference. It incorporates transcription dynamics into pseudotime inference while introducing lazy factors to mimic the effects of cell division. We compared LVPT with other methods in pseudotime inference using simulated datasets with different trajectory structures and demonstrated that our model had equivalent or better performance. We also applied LVPT to two real datasets of mouse pancreas and mouse hippocampus, and the pseudotime inferred by LVPT is consistent with prior knowledge, indicating that LVPT is an accurate and effective method for trajectory and pseudotime inference.

## Figures and Tables

**Table 1:** Performance comparison of LVPT with other methods in different structure databases. The values in the table represent the accuracy of different methods in different datasets.

Metrics	Structures	LVPT	VPT	VeTra	CellPath	PAGA	Monocle2	Slingshot	DPT	TSCAN
Correlation	Linear	**0.97**	0.94	0.92	0.92	0.90	0.96	0.88	0.82	0.96
	Bifurcating	**0.96**	0.93	0.92	0.91	0.87	0.89	0.79	0.78	0.64
	Trifurcating	**0.91**	0.89	0.87	0.88	0.85	0.82	0.73	0.68	0.67
HIM	Linear	**0.95**	0.94	0.89	0.92	0.93	0.91	0.90	0.79	0.83
	Bifurcating	**0.92**	0.85	0.73	0.62	0.86	0.79	0.83	0.70	0.60
	Trifurcating	**0.87**	0.83	0.64	0.70	0.82	0.65	0.78	0.56	0.52

## Data Availability

LVPT is written in Python and available at https://github.com/maoshuainan/lvpt (accessed on 10 August 2023).
